# WTAP regulates the production of reactive oxygen species, promotes malignant progression, and is closely related to the tumor microenvironment in glioblastoma

**DOI:** 10.18632/aging.205666

**Published:** 2024-03-25

**Authors:** Qiankun Ji, Yazhou Guo, Zibo Li, Xiaoyang Zhang

**Affiliations:** 1 Department of Neurosurgery, Zhoukou Central Hospital, Zhoukou 466000, Henan, P.R. China

**Keywords:** glioblastoma, reactive oxygen species, WTAP, cell proliferation, cell migration

## Abstract

RNA modifications have been substantiated to regulate the majority of physiological activities in the organism, including the metabolism of reactive oxygen species (ROS), which plays an important role in cells. As for the effect of RNA modification genes on ROS metabolism in glioblastoma (GBM), it has not been studied yet. Therefore, this study aims to screen the RNA modification genes that are most related to ROS metabolism and explore their effects on the biological behavior of GBM *in vitro*. Here, an association between WTAP and ROS metabolism was identified by bioinformatics analysis, and WTAP was highly expressed in GBM tissue compared with normal brain tissue, which was confirmed by western blotting and immunohistochemical staining. When using a ROS inducer to stimulate GBM cells in the WTAP overexpression group, the ROS level increased more significantly and the expression levels of superoxide dismutase 1 (SOD1) and catalase (CAT) also increased. Next, colony formation assay, wound healing assay, and transwell assay were performed to investigate the proliferation, migration, and invasion of GBM cells. The results showed that WTAP, as an oncogene, promoted the malignant progression of GBM cells. Functional enrichment analysis predicted that WTAP was involved in the regulation of tumor/immune-related functional pathways. Western blotting was used to identify that WTAP had a regulatory effect on the phosphorylation of PI3K/Akt signaling. Finally, based on functional enrichment analysis, we further performed immune-related analysis on WTAP. In conclusion, this study analyzed WTAP from three aspects, which provided new ideas for the treatment of GBM.

## INTRODUCTION

As the most malignant subtype of tumors in the central nervous system, GBM is characterized by invasive growth and high recurrence rate [1]. The standard treatment strategy for GBM includes extensive resection followed by post-operative concurrent radiotherapy with temozolomide, but all these treatments fail to obtain a satisfying curative effect, and the recurrence and prognosis of GBM remain dismal [2, 3]. Therefore, strengthening the basic research on the occurrence and development of GBM is still our priority.

ROS plays an important role in cells, including causing DNA damage and affecting the initiation and progression of tumors [4]. Studies have indicated that the ROS level has a significant effect on the efficacy of GBM-related chemotherapy drugs represented by temozolomide [5, 6]. Similarly, radiotherapy could destruct DNA structure and indirectly induce DNA damage to kill glioma cells by generating ROS. The metabolism of ROS also had a significant effect on the sensitivity of glioma cells to radiation [7, 8]. Therefore, ROS regulators may have an important impact on the prognosis of GBM. RNA modifications represented by N6-methyladenosine (m6A) have been substantiated to regulate the majority of physiological activities in the organism, including the metabolism of ROS in cells [9]. And such regulation of ROS metabolism has potential therapeutic implications for a variety of tumors, such as breast cancer and liver cancer [10, 11]. As for the effect of ROS metabolism regulated by RNA modifications in GBM has not been studied. By comparing the correlations of currently known 53 RNA modification-related enzymes with ROS metabolic pathways in public GBM databases, we screened out the 7 genes with the strongest correlation. Further comparing the difference in expression and prognostic significance of these genes in multiple glioma databases, we elected WTAP as the most significant gene associated with the ROS pathway.

As a subunit of methyladenine methyltransferase in m6A modification [12], WTAP plays a critical role in epi-transcriptomic regulation of RNA metabolism and is correlated with the occurrence and progression of multiple diseases, including liver disease, cardiovascular disease, and tumors [13–15]. Existing studies have shown that the aberrant higher expression of WTAP indicates a worse prognosis for various tumors [16–18]. And the specific mechanism may be that WTAP affects the immune infiltration of tumors [19, 20]. In glioma, the specific mechanism of high-WTAP leading to poor prognosis is still missing.

In this study, the U87 cell line stably overexpressed WTAP and the U251 cell line stably knocked down WTAP after lentivirus transfection were applied to perform cell function experiments compared with the control groups. Corresponding experimental results suggested that overexpression of WTAP had an obvious cancer-promoting effect in GBM. Combined with the results of GO and KEGG enrichment analysis in the GBM public databases, we found that WTAP was not only positively correlated with various oncogenic pathways but also closely related to immune-related pathways. Moreover, with the analysis results of GSEA software, we further conducted an experimental study on the regulatory relationship between WTAP and PI3K/AKT signaling pathway. Additionally, we used ssGSEA algorithm and GSVA algorithm to quantify the immune characteristics and immune cells. When the two algorithms were combined with the expression of WTAP to conduct a correlation analysis, we found that the expression of WTAP was significantly correlated with T cell infiltration. This conclusion was further verified by immunohistochemical experiments of WTAP and T cell-related markers. Our results unveiled that WTAP was a crucial potential prognostic biomarker of GBM.

Here, bioinformatics analysis indicates that WTAP is associated with ROS signature. Considering the importance of ROS in malignant tumors, we speculate that WTAP is involved in the regulation of ROS in GBM and may affect the malignant progression of GBM and the change of its tumor microenvironment. We hope that this study will provide a new target for the diagnosis and treatment of GBM.

## RESULTS

Bioinformatics analysis predicted a robust association between WTAP and ROS. The involvement of WTAP in regulating the level of ROS was determined in the GBM cells. In addition, it was measured that WTAP was upregulated in GBM tissues and promoted the malignant progression of GBM cells. Finally, the association between WTAP and TME was analyzed by bioinformatics analysis and verified simply by immunohistochemical staining.

### Exploration of the association between RNA modification genes and ROS signature

**Figure 1:**
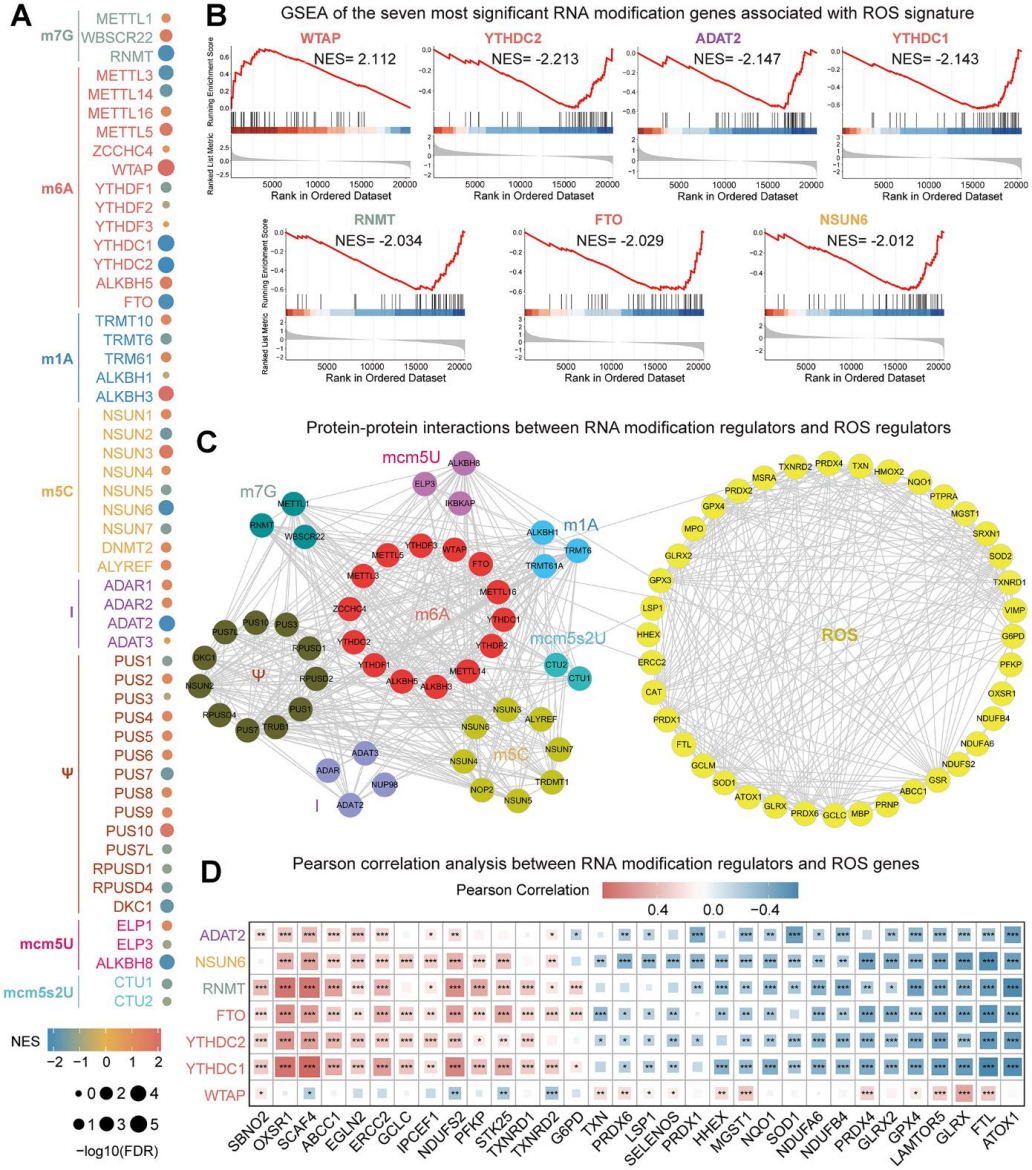
**Comprehensively analyze the association between RNA modification genes and ROS signature.** (**A**) Correlation between RNA modified genes and ROS signature. The size of the dot represents different FDR values, and the color of the dot represents different NES values. (**B**) Seven RNA modification genes most closely related to ROS signature were screened with an |NES| > 2 and FDR < 0.25. The line graph and gene sequencing list of the seven RNA modification genes were displayed. (**C**) Network diagram of the interaction between RNA modification regulators and ROS regulators. (**D**) Pearson correlation analysis between seven RNA modification genes and ROS genes. *P<0.05, **P<0.01, ***P<0.001.

Firstly, we explored the association between different types of RNA modification genes with ROS signature (Figure 1A). Seven RNA modification genes with an |NES| > 2 and FDR < 0.25 were screened (Figure 1B). The expression of WTAP was positively correlated with ROS signature, while the expression of YTHDC1, YTHDC2, ADAT2, RNMT, FTO, and NSUN6 was negatively correlated with it. Then, we explored the interaction between different types of RNA modification genes and ROS genes on the STRING website. The interaction data were further processed with Cytoscape software. The result showed that there was a close interaction between RNA modification genes, and the same phenomenon was also found between ROS genes. However, the interaction between RNA modification genes and ROS genes was very weak (Figure 1C). Finally, the correlation between seven RNA modification genes and ROS genes was explored. The result showed that there was a significant correlation between RNA modification genes and ROS genes (Figure 1D). The results showed that there was a significant correlation between these seven RNA modification genes and ROS genes. The correlation between WTAP and ROS genes is almost opposite to that of the other six RNA modification genes, which is consistent with the correlation analysis results between seven modification genes and ROS signature.

### Further screening of RNA modification genes

**Figure 2:**
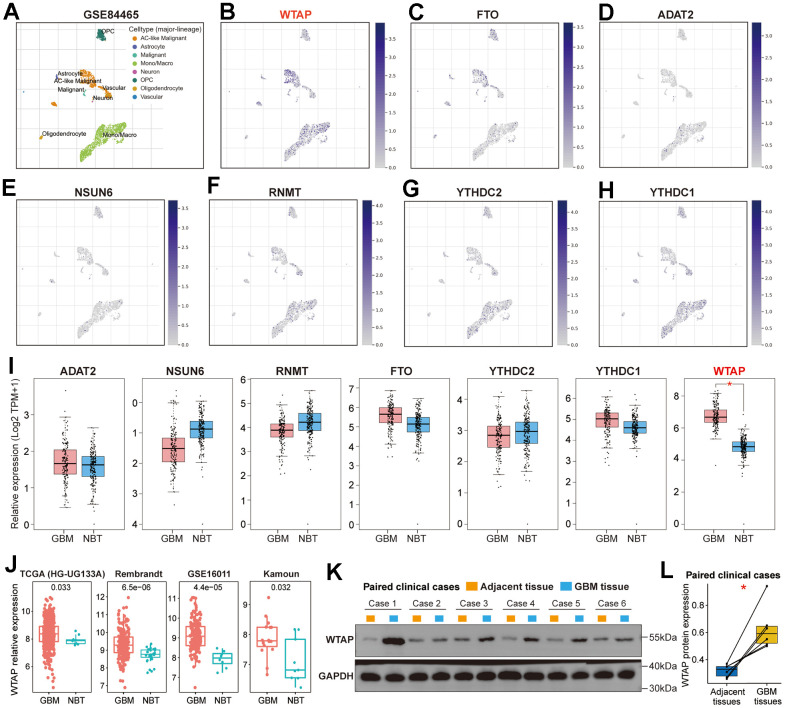
**Further screening of RNA modification genes.** (**A**) The scatterplot presented the eight kinds of cells with different colors in the TME of GBM based on the GSE84465 dataset. (**B**) The scatterplot presented the expression of WTAP in different types of cells. (**C**–**H**) The scatterplot presented the expression of FTO, ADAT2, NSUN6, RNMT, YTHDC2, and YTHDC1 in different types of cells. (**I**) Differential expression analysis of seven RNA modification genes between GBM tissues and NBTs. (**J**) The differential expression analysis of WTAP between GBM tissues and NBTs was performed in four independent GBM cohorts. (**K**) Western blot detection of the WTAP protein expression levels in paired GBM tissues and adjacent nontumor tissues. (**L**) Compared with adjacent nontumor tissues, WTAP protein expression was up-regulated in GBM tissues. *P < 0.05.

We explored the expression distribution of seven RNA-modification regulators in the GBM microenvironment on the TISCH website. According to the single-cell RNA sequencing data of the GSE84465 dataset, which contains 3,589 cells from four GBM patients, a total of eight main cell types were identified, including AC-like Malignant, Astrocyte, Malignant, Mono/Macro, Neuron, OPC, Oligodendrocyte and Vascular (Figure 2A). Compared with the other six RNA modification genes, WTAP has a higher expression level in TME, especially in AC-like malignant, monocytes and macrophages (Figure 2B–2H). Subsequently, we analyzed the differential expression of seven RNA modification genes between normal brain tissues and GBM tissues on the GEPIA website and found that only WTAP was differentially expressed (Figure 2I). In addition, significant differences in WTAP mRNA expression between normal brain tissues and GBM tissues were also found in four independent GBM cohorts (Figure 2J), and the number of samples in each cohort has been listed (Table 1). Furthermore, we extracted total protein from GBM tissues and corresponding adjacent tissues and conducted a western blot to detect the differential expression of WTAP protein. The results showed that WTAP protein was significantly up-regulated in GBM tissues compared with adjacent tissues (Figure 2K, 2L).

**Table 1:** Demographics and clinical information of GBM patients in TCGA (GDC), TCGA (HG-UG133A), CGGAseq-1, CGGAseq-2, Rembrandt, GSE16011, and Kamoun cohorts.

**Variables**	**Number**	**OS status**			**Histology**		
		**Alive**	**Dead**	**NA**	**GBM**	**NBT**	**NA**
**TCGA (GDC)**	Total (n=160)	31 (19.38%)	129 (80.63%)	0	160 (100%)	0	0
**TCGA (HG-UG133A)**	Total (n=538)	78 (14.50%)	447 (83.09%)	13 (2.42%)	528 (98.14%)	10 (1.86%)	0
**CGGAseq-1**	Total (n=249)	40 (16.06%)	198 (79.52%)	11 (4.42%)	249 (100%)	0	0
**CGGAseq-2**	Total (n=139)	13 (9.35%)	124 (89.21%)	2 (1.44%)	139 (100%)	0	0
**Rembrandt**	Total (n=247)	13 (5.26%)	190 (76.92%)	44 (17.81%)	219 (88.66%)	28 (11.34%)	0
**GSE16011**	Total (n=167)	11 (6.59%)	148 (88.62%)	8 (4.79%)	159 (95.21%)	8 (4.79%)	0
**Kamoun**	Total (n=25)	7 (28%)	8 (32%)	10 (40%)	16 (64%)	9 (36%)	0

GBM, glioblastoma; NBT, normal brain tissue.

### WTAP is upregulated in GBM tissues

**Figure 3:**
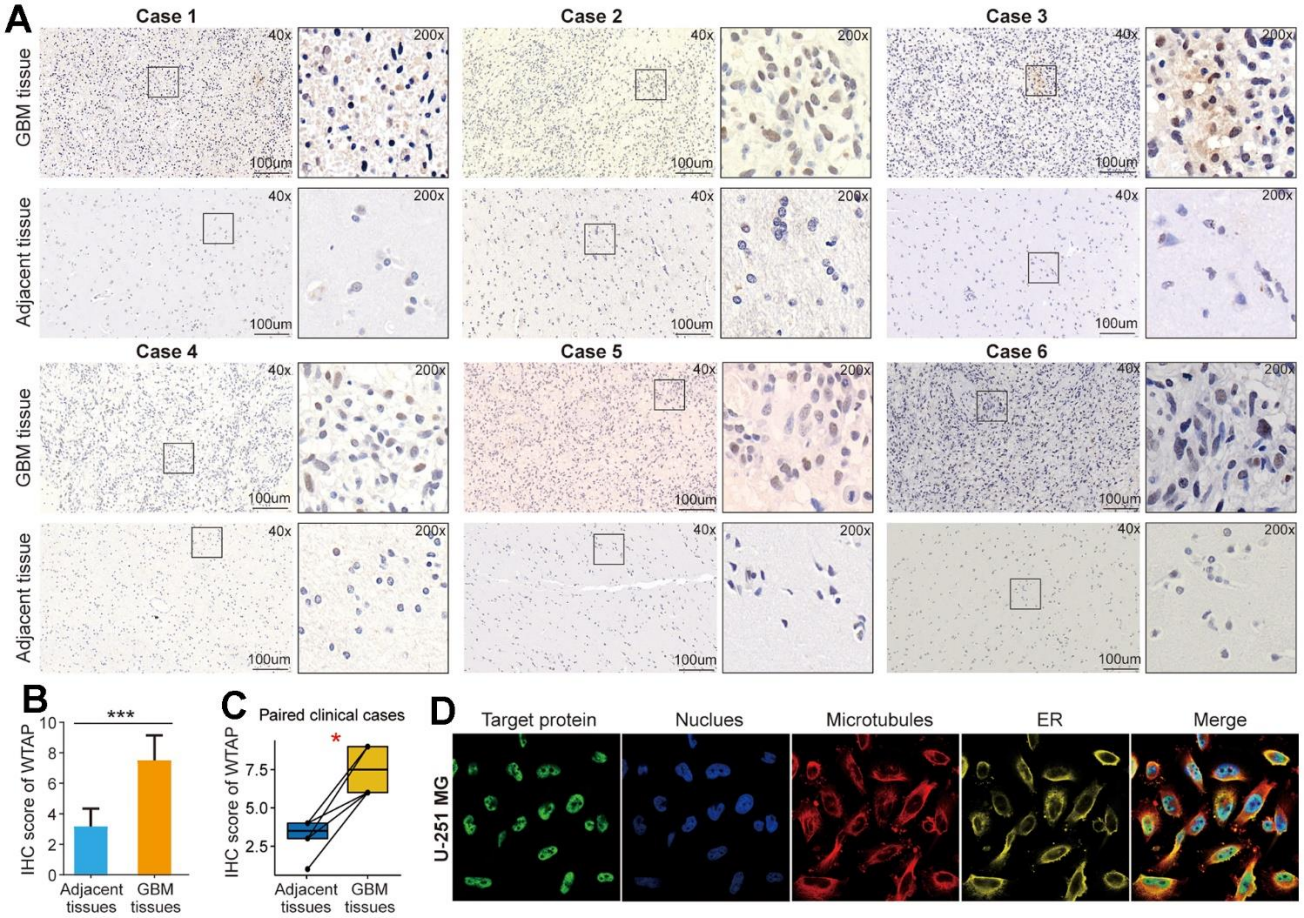
**WTAP protein is upregulated in GBM tissues.** (**A**) An immunohistochemistry assay was used to analyze the differential expression of WTAP protein in six pairs of GBM tissues and adjacent nontumor tissues. (**B**) The IHC scores of WTAP in GBM tissues were significantly higher than that of matched adjacent nontumor tissues. (**C**) Immunohistochemical detection of the WTAP expression levels in paired GBM tissues and adjacent nontumor tissues. (**D**) The immunofluorescence images of WTAP protein, nucleus, microtubules, and endoplasmic reticulum (ER) in U251 MG cell. *P < 0.05; ***P < 0.001.

The tissue samples of GBM were obtained from patients who underwent surgical treatment in Zhoukou Central Hospital. IHC staining was used to detect the differential expression of WTAP protein between GBM tissues and corresponding adjacent tissues. Representative IHC images showed that WTAP protein expression was significantly up-regulated in GBM tissues compared with corresponding adjacent tissues (Figure 3A–3C). In addition, we found that in GBM tissues, WTAP protein was mainly localized in the nucleus. The immunofluorescence images of the U251 cell searched from the HPA website showed that WTAP protein was mainly located in the nucleus, and its protein expression can hardly be seen in other cell organs (Figure 3D). The above studies demonstrate that WTAP is up-regulated in GBM tissues and mainly located in the nucleus, which is not only conducive to the role of RNA modification of WTAP but also conducive to its participation in the regulation of GBM progress.

### Survival analysis of seven RNA modification genes

**Figure 4:**
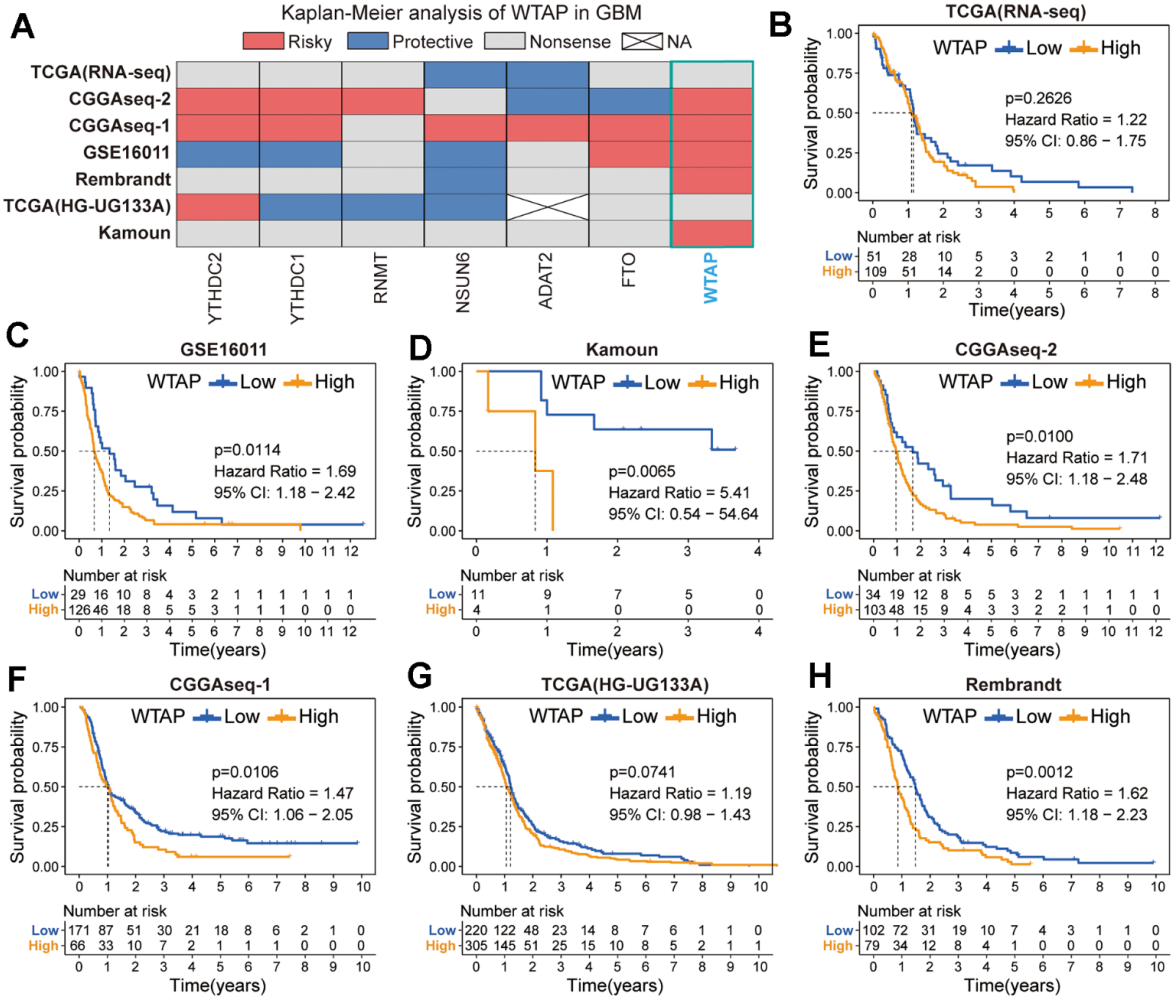
**Prognostic analysis of seven RNA modification genes.** (**A**) Summary of the prognostic value of seven RNA modification genes in seven independent GBM cohorts. Red represents a prognostic risk factor, blue indicates a beneficial factor, and grey represents a p-value > 0.05. (**B**–**H**) Kaplan-Meier prognostic curves of WTAP in TCGA (RNA-seq) (**B**), GSE16011 cohort (**C**), Kamoun (**D**), CGGAseq-2 (**E**), CGGAseq-1 (**F**), TCGA (HG-UG133A) (**G**) and Rembrandt (**H**) cohorts.

Based on the results of differential expression analysis, we then explored the prognostic value of these seven RNA modification genes. The heatmap contains the Kaplan-Meier survival analysis results of WTAP, FTO, ADAT2, NSUN6, RNMT, YTHDC1, and YTHDC2 based on seven independent GBM cohorts, which are TCGA (RNA-seq), TCGA (HG-UG133A), CGGAseq-1, CGGAseq-2, Kamoun, GSE16011, and Rembrandt cohorts (Figure 4A and Table 1). We found that the prognostic value of the other six RNA modification genes was not stable except for WTAP. In addition to TCGA (RNA-seq) and TCGA (HG-UG133A) cohorts, WTAP can be used as a prognostic risk factor to predict the prognosis of GBM patients effectively in the other five GBM cohorts (Figure 4B–4H).

### WTAP promotes ROS production

**Figure 5:**
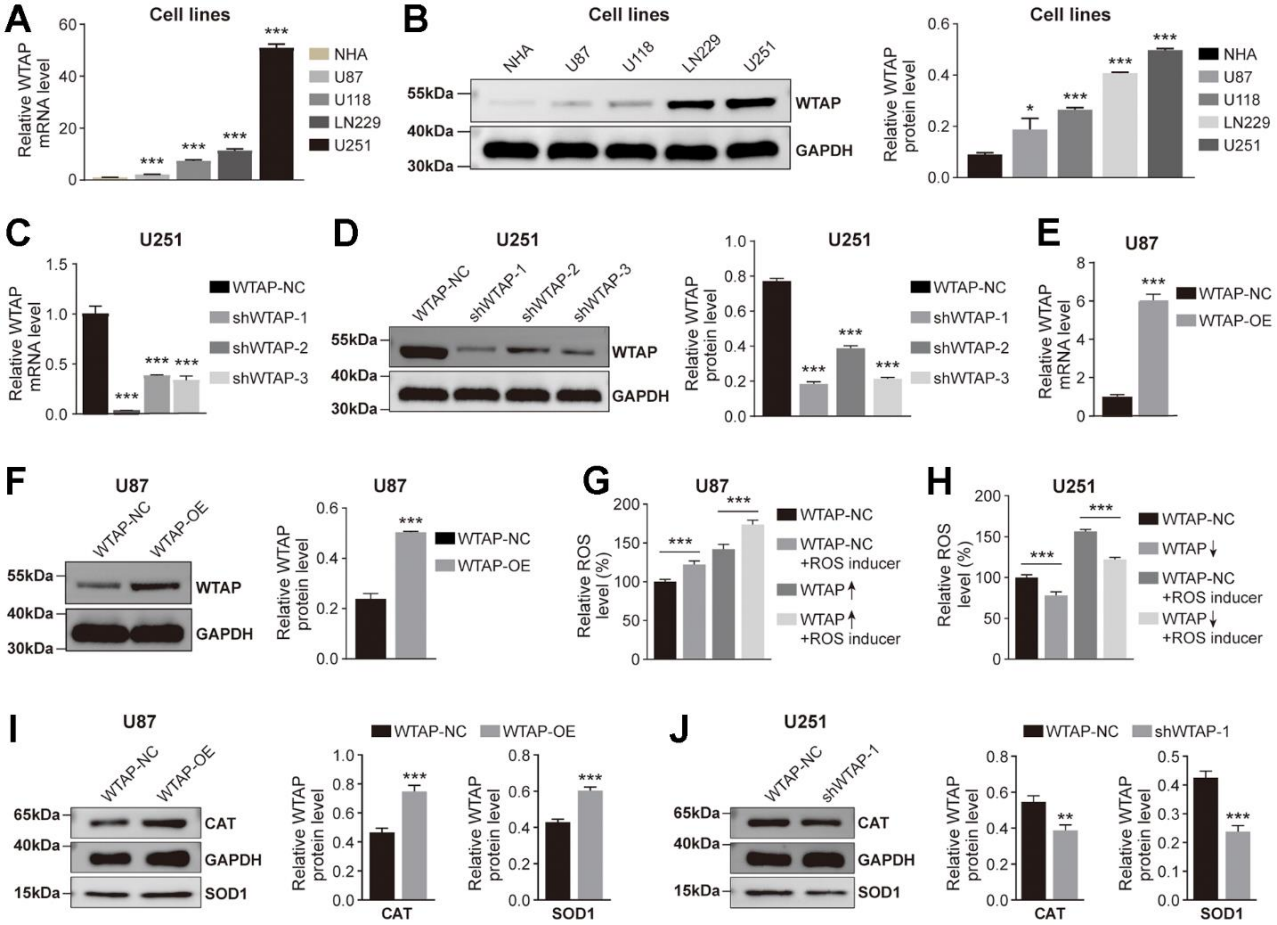
**WTAP promotes ROS production.** (**A**, **B**) RT-PCR (**A**) and western blotting (**B**) were used to analyze the differential expression of WTAP among NHA, U87, U118, LN229, and U251 cell lines. (**C**, **D**) The knockdown efficiency of shWTAP-1, -2, and -3 in U251 cells was analyzed by RT-PCR (**C**) and western blotting (**D**). shWTAP-1 was chosen in subsequent experiments because of its outstanding efficiency in silencing WTAP. (**E**, **F**) The overexpression efficiency of WTAP in U87 cells transfected with lentivirus was analyzed by RT-PCR (**E**) and western blotting (**F**). (**G**, **H**) The effect of the change of WTAP expression level on ROS production in U87 (**G**) and U251 (**H**) cells was detected by a fluorescent microplate reader. (**I**, **J**) WTAP participates in the regulation of CAT and SOD1 protease expression in U87 (**I**) and U251 (**J**) cells. *P < 0.05; **P < 0.01; ***P < 0.001.

Based on the above research results, we then explored whether WTAP participates in regulating the production of ROS in GBM cell lines. The mRNA and protein abundance of WTAP in GBM cell lines were significantly upregulated compared with the NHA cell line (Figure 5A, 5B). Among the four GBM cell lines, U87has the lowest endogenous WTAP expression, and U251 has the highest endogenous WTAP expression. therefore, U87 and U251 were used to establish the stably overexpressed and silenced WTAP cell lines, respectively. Subsequently, WTAP over-expressed lentivirus (WTAP-OE) and three independent WTAP shRNA-expression lentivirus (shWTAP-1, shWTAP-2, and shWTAP-3) were constructed, and the overexpression or knock-down effects were examined by RT-qPCR and immunoblotting assays (Figure 5C–5F). Finally, we explored whether WTAP participated in the regulation of ROS production in GBM cell lines through *in vitro* experiments. The results showed that whether or not a ROS inducer was added, in U87 cell line, the level of ROS in the control group of WTAP was lower than that in the high expression group (Figure 5G). And in U251 cell line, the level of ROS in the control group of WTAP was higher than that in the low expression group (Figure 5H). In addition, we also detected the expression level of ROS-related regulatory enzymes in U87 and U251 cell lines. The results showed that the expression levels of SOD1 and CAT decreased in the low expression group of WTAP, but increased in the high expression group of WTAP (Figure 5I, 5J). The above results suggest that WTAP may affect ROS production by regulating the expression of ROS-related regulatory enzymes.

### WTAP enhances the malignant biological behavior of GBM cells

**Figure 6:**
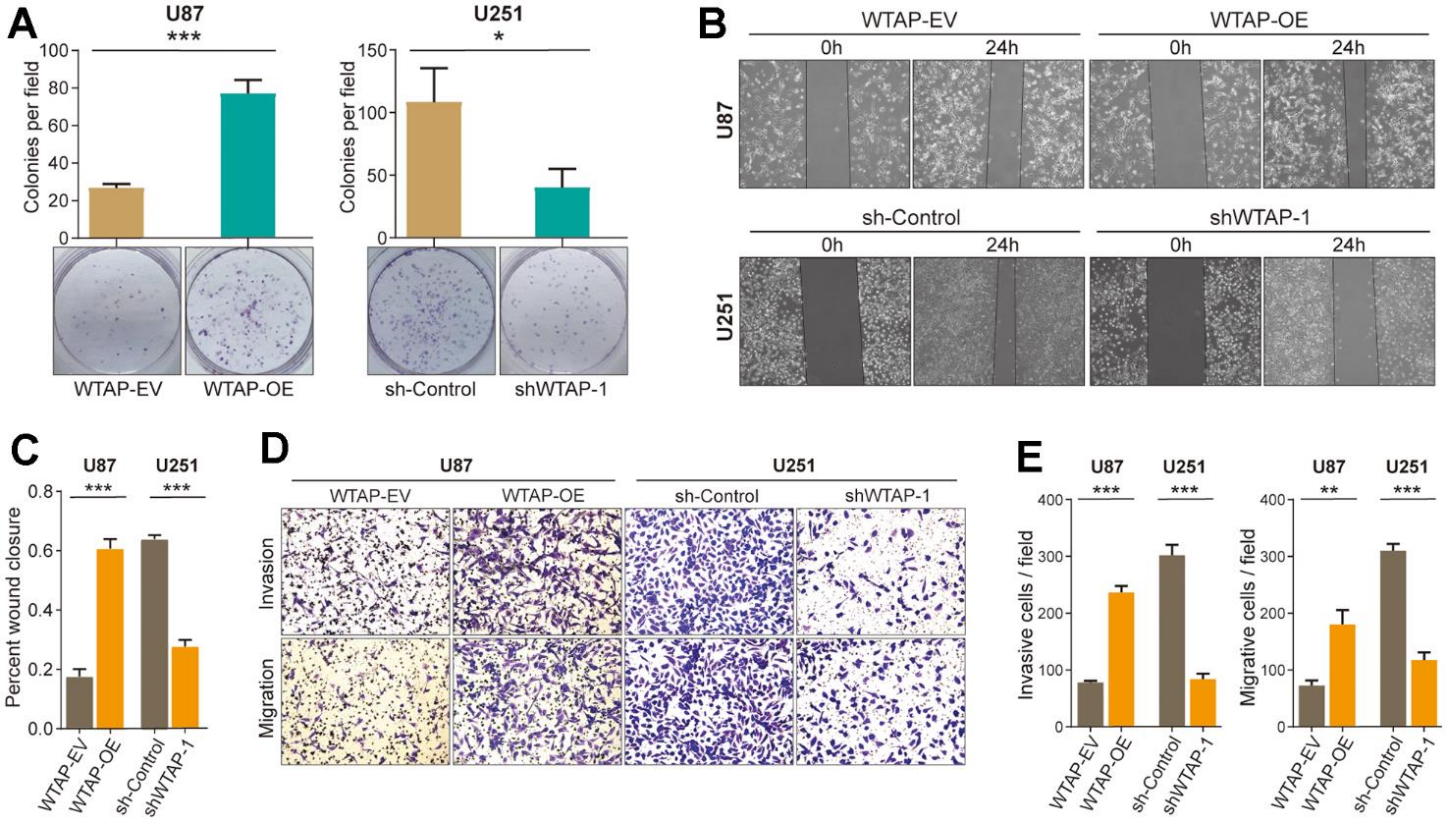
**WTAP promotes the malignant progression of glioblastoma cell lines.** (**A**) A colony formation assay was used to explore the effect of WTAP on the proliferation ability of U87 and U251 cells. (**B**, **C**) A wound-healing test was used to determine the effect of WTAP on the migration ability of U87 and U251 cells. (**D**, **E**) The invasion ability and migration ability were measured by transwell assay. *P < 0.05; **P < 0.01; ***P < 0.001.

Some studies reported that ROS was involved in regulating the progression of tumors [4]. Therefore, we will study whether WTAP affects the malignant progression of GBM *in vitro*. The colony formation assay (Figure 6A) was conducted to determine the effects of WTAP on the proliferation ability of GBM cells. A wound healing test (Figure 6B, 6C) and transwell assay (Figure 6D, 6E) were performed to detect the migratory and invasive abilities of GBM cells. Compared with the control groups, the overexpression of WTAP distinctly enhanced the proliferation, migration, and invasion ability of U87 cells. In contrast, knockdown of WTAP expression markedly inhibited the proliferation, migration, and invasion ability of U251 cells.

### Function enrichment analysis of WTAP in GBM

**Figure 7:**
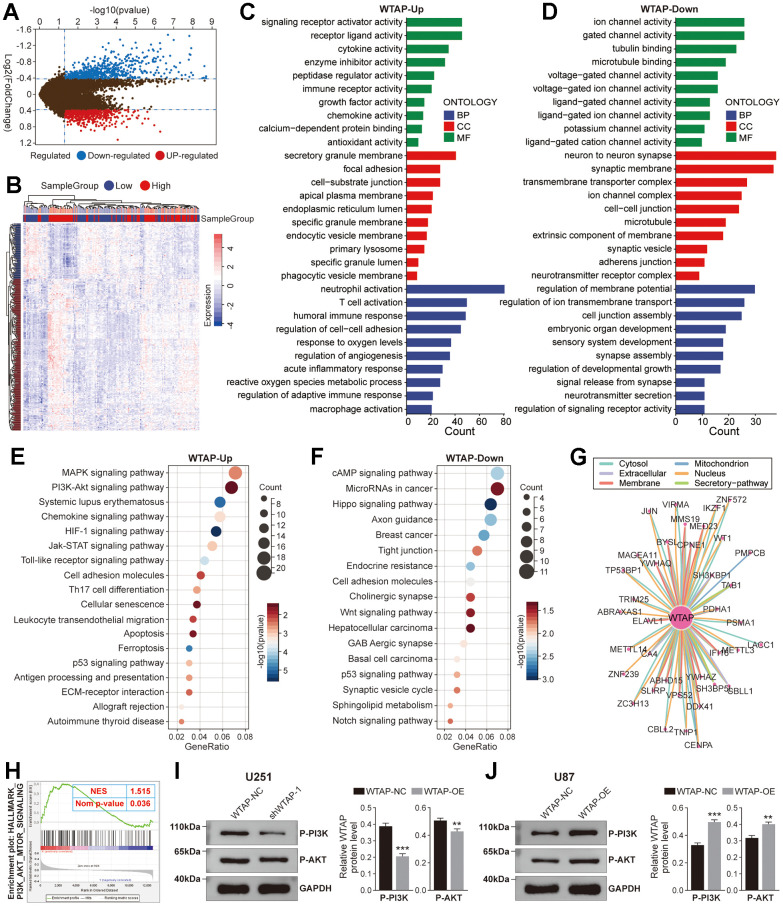
**Function enrichment analysis of WTAP.** (**A**) The volcano plot showed differentially expressed genes (DEGs) based on the median value of WTAP expression in the TCGA cohort. (**B**) The heat map showed the relative expression levels of 1180 DEGs between WTAP high and low expression groups. (**C**) Enrichment analysis for GO term of up-regulated genes. (**D**) Enrichment analysis for GO term of down-regulated genes. (**E**) Enrichment analysis for KEGG pathway of up-regulated genes. (**F**) Enrichment analysis for KEGG pathway of down-regulated genes. (**G**) The PPI network showed the proteins that interact most closely with WTAP. (**H**) According to GSEA, the high expression of WTAP in the PI3K/AKT/mTOR signaling pathway was enhanced from the TCGA GBM dataset. (**I**, **J**) WTAP participates in the regulation of P-PI3K and P-AKT expression in U251 (**I**) and U87 (**J**) cells. *P < 0.05; **P < 0.01; ***P < 0.001.

Although the effect of WTAP on the biological behavior of GBM cell lines has been verified *in vitro*, it is still unclear which functional pathways WTAP is mainly involved in. Therefore, we performed a gene enrichment analysis on WTAP. 1180 DEGs were recognized between high- and low-WTAP subgroups in the TCGA GBM dataset, containing 543 positive associated genes and 637 negative associated genes (Figure 7A, 7B). GO enrichment analysis revealed that the positively associated genes were significantly enriched in receptor activity-related pathways and immune-related biological processes, such as receptor-ligand activity, immune receptor activity, T cell activation, neutrophil activation, and macrophage activation (Figure 7C), while negatively associated genes were enriched in synaptic transmission-related processes, like ligand-gated channel activity, ion channel activity and synapse assembly (Figure 7D). KEGG pathway analysis revealed that the positively associated genes were closely related to the PI3K-Akt signaling pathway, chemokine signaling pathway, HIF-1 signaling pathway, and cell adhesion molecules (Figure 7E), while down-regulated genes were closely associated with the cAMP signaling pathway, MicroRNAs in cancer and WNT signaling pathway (Figure 7F). The PPI data of WTAP obtained from the ComPPI website was used to draw the PPI network diagram, which displayed the proteins closely related to WTAP at different subcellular structures, including cytosol, mitochondrion, nucleus, extracellular, membrane, and secretory-pathway (Figure 7G). In addition, it was found that there was an obvious positive correlation between WTAP and PI3K/AKT/mTOR signaling with an NES = 1.515 and normal p-value = 0.036 (Figure 7H). Phosphorylated PI3K and Akt represent the activation of PI3K and Akt. Western blot shows that knockdown of WTAP expression can inhibit the expression of P-PI3K and P-Akt, and overexpression of WTAP can promote the expression of P-PI3K and P-Akt (Figure 7I, 7J).

### Immune-related analysis

**Figure 8:**
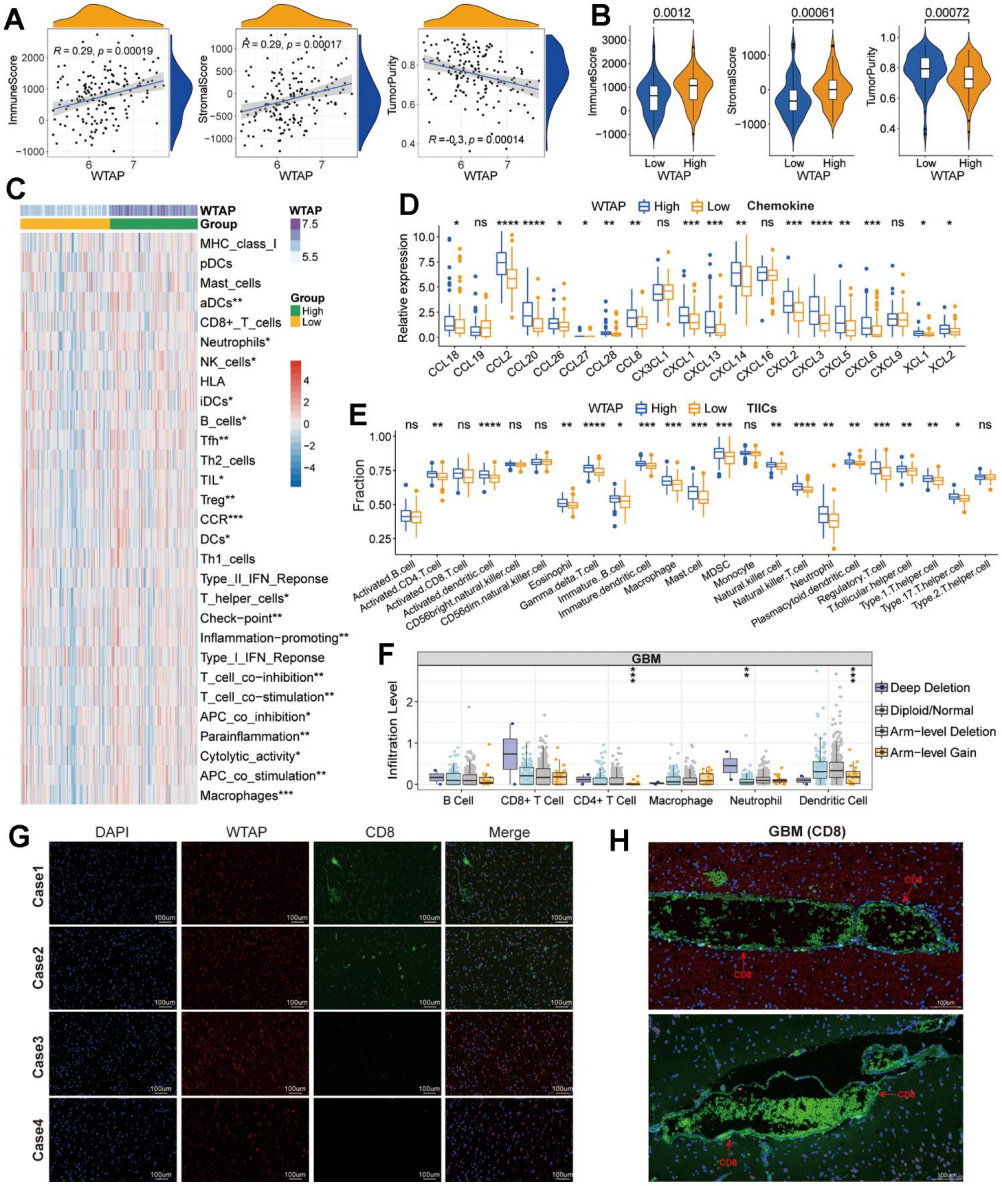
**Immune-related analysis.** (**A**) Correlation between WTAP and immune score, stromal score, and tumor purity. (**B**) Difference analysis of TME-related scores between low and high WTAP expression groups. (**C**) Difference analysis of enrichment scores of immune-associated gene sets between low and high WTAP expression groups. (**D**) Differential expression analysis of chemokines between low and high WTAP expression groups. (**E**) The difference in immune infiltration level of TIICs between low and high WTAP expression groups in TCGA cohort. (**F**) Copy number variation of WTAP affects the infiltrating levels of CD4+ T cells, neutrophils, and dendritic cells. (**G**) Immunofluorescence staining of the nucleus, WTAP, and CD8 in four cases of GBM tissues. (**H**) Immunofluorescence staining of blood vessels and perivascular areas of GBM tissues. *P<0.05, **P<0.01, ***P<0.001, ****P<0.0001, ns, nonsense.

The results of GO and KEGG enrichment analysis remind us that WTAP may be involved in the regulation of immune-related functional processes. Therefore, we subsequently explored the association between WTAP expression and immune-cell infiltration in GBM based on the TCGA GBM dataset. Immune score, stromal score, and tumor purity represent the content of immune cells, stromal cells, and tumor cells in the TME respectively, which has a significant correlation with prognosis [21]. Scatter plots and violin diagrams suggested that there is a positive correlation between WTAP and immune-score and stromal-score, and a negative correlation between WTAP and tumor purity (Figure 8A), and there were significant differences in the three types of immune-related scores between low and high WTAP expression groups (Figure 8B). Then, we calculated the enrichment scores of immune signatures with the ESTIMATE algorithm and found that the enrichment scores of most immune signatures were higher in the high expression group (Figure 8C), which means that the immune activity of the WTAP high expression group is higher than WTAP low expression group. Chemokines play an important role in guiding immune cell migration, which is necessary to initiate and transmit an effective anti-tumor immune response. The secretion of chemokines in TME usually changes, which affects the differentiation and infiltration of immune cells in TME [22]. Compared with the low WTAP expression group, the expression level of most chemokines in GBM with high WTAP expression tend to be high (Figure 8D), which promoted the change of TME in the WTAP-high expression group. Subsequently, the content of 23 types of immune cells was calculated by the ssGSEA algorithm. Except for activated B cell, activated T cell, CD56+/- natural killer cell, monocyte, and type2 T helper cell, the content of other types of immune cells is significantly higher in the WTAP-high expression group when compared with the WTAP-low expression group (Figure 8E). In addition, the CNV of WTAP also affects the infiltration levels of CD4+ T cells, neutrophils, and dendritic cells in GBM (Figure 8F). Given the importance of CD8+ T cells in tumor immunotherapy, we further explored the correlation between WTAP and CD8+ T cells in four cases of GBM tissues. We observed that the expression of CD8 protein was low or even not expressed in GBM tissues (Figure 8G). However, CD8 protein was significantly increased in the capillary wall and capillary interior (Figure 8H). These results may be directly related to the inability of immune cells to effectively pass through the blood-brain barrier and can explain the poor effect of immunotherapy on GBM to some extent.

## DISCUSSION

The effect of oxidative stress in tumors is determined by the concentration of ROS, which can be both tumor-promoting and tumor-suppressing [4]. In addition to the dual effect on tumor cells, ROS can also affect the drug resistance of chemotherapy [23] and radiotherapy sensitivity, specifically for glioma [24]. Therefore, the regulation of ROS may have a more significant impact on the prognosis of glioma than other tumors. Based on this hypothesis, we used bioinformatics methods to analyze the public database of GBM and screened WTAP as our target gene. As one of the seven genes with the most obvious correlation to the ROS signature in RNA modification-related genes, the expression of WTAP also had strong prognostic significance in GBM.

To verify the expression of WTAP in GBM, we selected six GBM tissues and corresponding adjacent tissues for immunofluorescence, WB, and PCR experiments. The results showed that the expression of WTAP in GBM tissues was significantly increased. We further used U87 and U251 cell lines as experimental models, and transfected lentiviruses to stably knockdown and overexpressed WTAP for ROS level detection experiments and functional experiments. The results suggested that the expression of WTAP can increase ROS levels and promote the monoclonal, migration, and invasion abilities of the GBM cell line. The results were consistent with our hypothesis and previous research conclusions [18].

To further study the specific mechanism of cell function changes caused by WTAP, we performed the GO and KEGG functional enrichment analysis based on the TCGA database. The results indicate that high expression of WTAP was associated with a variety of classical cancer-promoting pathways including the MAPK signaling pathway and PI3K-Akt signaling pathway, which had been proved to be the ROS-related mechanism of promoting tumor growth [25]. In addition, the high expression of WTAP was also accompanied by increased immune-related pathways. Now immunotherapy has become an important adjuvant therapy for cancers [26], but its effect on glioma was still unsatisfactory [27]. We tried to further study the immune infiltration characteristics of glioma by analyzing the function of WTAP.

In our subsequent analysis results, WTAP and infiltration level of immune cells were significantly correlated, and the gene copy number variation of WTAP could affect the infiltration level of immune cells. In the correlation analysis with 29 representatives of “immune signatures”, the expression of WTAP was positively correlated with most immune signatures, which is consistent with the results of the functional enrichment analysis. Specifically, the expression of WTAP was also positively correlated with the various chemokines and the percentage of immune cells. The above results suggest that WTAP may be extensively involved in the regulation of immune infiltration in GBM. Interestingly, in functional enrichment analysis results, we found high WTAP expression raised the T cell activation pathway and Th17 cell differentiation pathway. But in the comparison of the quantity among different immune cell types, activated CD4+ T cells had a significant difference between the subgroups, and activated CD8+ T cells showed no difference. To verify the results of the bioinformatics analysis, we performed fluorescence immunohistochemistry experiments on GBM tissue sections for WTAP and CD8+ T cell marker (CD8). CD8+ T cells were significantly aggregated in the vascular wall and interior of GBM tissues, but the infiltration level in perivascular tissues was low and had no obvious association with WTAP, which indicates that WTAP cannot effectively induce CD8+ T cell infiltration. The assumption may be that CD8+ T cells are difficult to cross the blood-brain barrier.

In conclusion, the expression of WTAP can significantly affect the prognosis of GBM patients and can be used as a potential prognostic biomarker for GBM. In particular, the correlation between WTAP expression and TME of GBM may be one of the reasons causing the poor prognosis of patients with high WTAP expression. However, this study also has limitations. We cannot fully demonstrate whether WTAP promoted the development of GBM immune infiltration via the regulation of ROS. Moreover, ROS levels also regulate the expression and activity of m6A enzymes [9]. The mechanisms regulating the ROS pathway via WTAP-mediated RNA modification will be our next research direction.

## CONCLUSIONS

This study initially explored the relationship between WTAP and ROS in GBM and verified that WTAP plays a role in oncogene in GBM. In addition, WTAP is closely related to the TME of GBM, which may be involved in regulating the secretion of chemokines and the migration of immune cells in TME. In general, WTAP is of great significance for the diagnosis and prognosis of GBM and is expected to become a new therapeutic target for GBM.

## MATERIALS AND METHODS

### Data source

The transcriptomic data and corresponding clinicopathological information of GBM patients were downloaded from public datasets. TCGA (RNA-seq), TCGA (HG-UG133A), CGGAseq-1, CGGAseq-2, Kamoun, GSE16011, and Rembrandt cohorts were downloaded from the GDC database (https://portal.gdc.cancer.gov/) and GlioVis website (http://gliovis.bioinfo.cnio.es/). Single-cell sequencing analysis of glioma from the GSE84465 dataset was performed in the Tumor Immune Single-cell Hub [28]. The immunofluorescent staining images of the expression and distribution of WTAP protein in U-251 MG were obtained from The Human Protein Atlas (HPA) database [29].

### Gene set enrichment analysis

The hallmark gene set “REACTIVE_OXYGEN_SPECIES_PATHWAY” was extracted from the Molecular Signatures Database. The association between RNA modification genes and ROS gene set was calculated by using the R package “clusterProfiler” [30], and the normalized enrichment score (NES) and false discovery rate (FDR) were displayed in the bubble plot. If the GSEA result with the criteria of |NES| > 2 and FDR < 0.25, the corresponding line graph and gene sequencing list will be displayed by using the R package “ggplot2”.

### Differential expression analysis and prognosis analysis of seven RNA modification genes

The differential expression of seven RNA modification genes in normal brain tissues and GBM tissues was analyzed on the GEPIA website [31]. Then, the differential expression of WTAP was further analyzed in four independent GBM cohorts using the R package “ggpubr”. The prognostic roles of the seven RNA modification genes were evaluated with the Kaplan–Meier method and presented in the heatmap. The Kaplan–Meier survival curves of WTAP with optimal cutoff value, statistically significant log-rank p-value, hazard ratio (HR), and 95% confidence interval (CI) in the four independent GBM cohorts were drawn by using the R packages “survival” and “survminer”.

### Cell culture and transfection

The Chinese Academia Sinica Cell Repository in Shanghai provided us with the cell lines needed for this study, including U87, U118, LN229, U251, and immortalized Normal human astrocyte (NHA). The cell culture conditions and procedures were described in our previous studies [32]. The WTAP knockdown and overexpression lentivirus were constructed by the Sheweisi Biotechnology Company (Tianjin, China) using hU6-MCS-CBh-gcGFP-IRES-puromycin. The sequence of WTAP shRNA is as follows: shWTAP-1, 5ʹ-ccGCAAGTACACAGATCTTAACT-3ʹ, shWTAP-2, 5ʹ-ccGCCCAACTGAGATCAACAATG-3ʹ, and shWTAP-3, 5ʹ-ccGATTGAGTGAAACAGACTTCA-3ʹ. For lentivirus transfection, refer to the instructions provided by the Sheweisi Biotechnology Company.

### qRT-PCR and western blot

The primer sequences of WTAP and GAPDH (internal reference gene) purchased from Ribobio (Guangzhou, China) are as follows: WTAP F primer: 5’-GGAAAGGACGGGGAGTGTTAC-3’, R primer: 5’-GCATTCGACACTTCGCCATT-3’; GAPDH F primer: 5’-TGTGGGCATCAATGGATTTGG-3’, R primer: 5’-ACACCATGTATTCCGGGTCAAT-3’. The WTAP rabbit polyclonal antibody (GB111531, Servicebio, China), catalase polyclonal antibody (21260-1-AP, Proteintech, China), SOD1 polyclonal antibody (10269-1-AP, Proteintech), Anti-p-PI3K (#4228S, CST), phospho-AKT polyclonal antibody (28731-1-AP, Proteintech), and mouse monoclonal to GAPDH (GB15002, Servicebio) were used as primary antibodies. Goat anti-rabbit IgG (GB23303, Servicebio) and goat anti-mouse IgG (GB25301, Servicebio) were the second antibodies. For the dilution ratio of antibodies, refer to the corresponding instructions. The extraction methods of cell protein and RNA, as well as the operation methods of western blotting and qRT-PCR, are completely consistent with that described in our previous study [32]. In addition, we also extracted tissue proteins in this study, and the extraction methods are as follows. First, tissue was weighed and cut, and put into a 2ml EP tube. RIPA and PMSF were mixed in the proportion of 100:1 to prepare tissue lysate. Then add the lysate into the tissue according to the ratio of adding 1 ml lysate per 50mg of tissue, homogenized with a homogenizer, and then lysed on ice for 30 minutes. Finally, the tissue was centrifuged for 10 minutes with a low-temperature high-speed centrifuge at 4° C and 12000 rpm. The subsequent steps are the same as the extraction of cellular proteins.

### Immunohistochemical and immunofluorescence staining

Six pairs of GBM and adjacent samples were fixed with 10% formalin for one week. The tissues were embedded with paraffin and sectioned (four-micrometer) subsequently. Next, the tissue sections were deparaffinized and dehydrated, and treated with 3% hydrogen peroxide for about 10 minutes. Primary antibodies against WTAP (60188-1-Ig, Proteintech) were used to stain tissues at 4 C for one night after blocking with 5% BSA for about one hour at RT. Then, treated with corresponding secondary antibodies for one hour at RT. Then DAB staining, target molecules detection, and hematoxylin counterstaining in turn. For immunofluorescent staining, tissue sections were immunostained with primary antibodies against WTAP and CD8 (66868-1-Ig, Proteintech) overnight at 4° C, and then incubated with fluorochrome-conjugated antibodies. After that, DAPI was added as a nuclear counterstain. A fluorescence microscope was used to capture images.

### Colony formation assay

For colony formation assay, we inoculated 600 cells in each well of the 6-well plate and added 3 ml of complete medium to each well. Subsequently, we checked the cell growth status and replaced the medium every three days. Culture for about two weeks, and terminate the culture when the number of cells in most colonies is more than 30. After fixing with formaldehyde and dyeing with crystal violet, taking photos and counting the number of cell colonies were done with ImageJ software.

### Wound healing assay

When the cells in the 6-well plate reached complete confluence, the medium was removed and a scratch was conducted by using a 200 μl of the sterile pipette tip. The 6-well plates were washed with PBS and added with a serum-free medium for further culture. Images were captured at 0 and 24 h respectively. After measuring the original and actual wound areas, we calculate the percentage of wound closure.

### Transwell assay

For the invasion assay, the Matrigel matrix was evenly spread at the bottom of the transwell chamber of 24-well plates (Corning, USA) and then incubated at 37 C for about 5 h to ensure that the Matrigel matrix solidifies. GBM cells (1 × 10^5^) were added to the upper chamber with a serum-free medium, and the lower chamber was added with a medium containing 10% FBS. After incubation for about 6 h, the invasion cells that cross the transwell membrane were stained with crystal violet. Finally, take photos with a microscope and count the cells with ImageJ software. All experiments were conducted in triplicate.

### Detection of ROS and related regulated enzymes

The probe was loaded onto the adherent cells *in situ* according to the instructions of the ROS assay kit. 2’,7’-dichlorofluorescin diacetate (DCFH-DA; Beyotime, S0033M, China) was diluted with the serum-free medium at a ratio of 1:1000, then added to U87 and U251 cells and incubated in 37° C incubators for 20 minutes. Then, the cells were washed three times with a serum-free medium to fully remove DCFH-DA that did not enter the cells. Then ROS inducer (BestBio, BB-47058, China) was added to induce the oxidative stress response of cells. Finally, ROS was detected by a fluorescent microplate reader. In addition, we also detected the differential expression of ROS-related regulatory enzymes CAT (21260-1-AP, Proteintech) and SOD1 (10269-1-AP, Proteintech) between different groups by western blot.

### Function enrichment analysis based on TCGA-GBM cohort

According to the mRNA expression level of WTAP, GBM patients were divided into WTAP-high and WTAP-low groups. The R package “limma” was utilized to screen the differentially expressed genes (DEGs) between high- and low-WTAP expression groups with the threshold: log2 fold change ≥ 1.3 (up-regulated) or log2 fold change ≤ −1.3 (down-regulated) [33]. Then, we conducted Gene Ontology (GO) and Kyoto Encyclopedia of Genes enrichment analysis to find the potential functional annotation of these DGEs in GBM using the R package “clusterProfiler” [30].

### Immune infiltration analysis

The content of immune cells and stromal cells in TME is of great significance to tumor progression and immunotherapy [34]. The ESTIMATE algorithm can effectively calculate the tumor purity, stromal score, and immune score to evaluate the content of immune cells and stromal cells in the TME [35]. The R packages “ggpubr” and “ggExtra” were used to analyze the association between TME-related scores and WTAP. We used ssGSEA to calculate the content of TIICs and the enrichment scores of immune signatures, which are known to be closely related to tumor immune activity [36]. Tumor IMmune Estimation Resource (TIMER 2.0) was used to explore the effect of copy number variation (CNV) of WTAP on the infiltrating levels of immune cells in GBM [37].

### Statistical analysis

The statistical analysis was conducted with GraphPad Prism (version 8.0.2). Spearman correlation analyses were used to explore correlations between two groups of variables. The Wilcoxon rank-sum test was used to compare two groups or categories; for more than two groups or categories, the Kruskal–Wallis test was used. Survival analysis was assessed by K–M method with the log-rank test. *P* < 0.05 (*) was considered statistically significant.

### Availability of data and material

Data associated with this study are summarized in the manuscript. The Cancer Genome Atlas (TCGA) database: https://portal.gdc.cancer.gov/. Data Visualization Tools for Brain Tumor Datasets (GlioVis): http://gliovis.bioinfo.cnio.es/. Molecular Signatures Database: https://www.gsea-msigdb.org/gsea/index.jsp. Tumor Immune Single-cell Hub (TISCH) database: http://tisch.comp-genomics.org/documentation/. The Human Protein Atlas (HPA) database: https://www.proteinatlas.org/. Gene Expression Profiling Interactive Analysis (GEPIA) database: http://gepia.cancer-pku.cn/detail.php. Tumor IMmune Estimation Resource (TIMER) database: https://cistrome.shinyapps.io/timer/. Compartmentalized Protein-Protein Interaction database (ComPPI): http://comppi.linkgroup.hu/protein_search/interactors/O 15197.
